# Cold compressive and tribological properties of forged and heat-treated Ti-6Al-3Mo-2Sn-2Zr-2Nb-1.5Cr-0.1Si alloy

**DOI:** 10.1038/s41598-025-32468-y

**Published:** 2026-01-07

**Authors:** Ramadan N. Elshaer

**Affiliations:** Tabbin Institute for Metallurgical Studies (TIMS), Cairo, Egypt

**Keywords:** TC21 Ti-alloy, Forging, Cold compressive, Tribological, Worn surface texture, Abbott Firestone curve, Engineering, Materials science

## Abstract

The impact of forging and heat treatment processes on cold compressive and tribological properties (wear rate & friction coefficient) of Ti-6Al-3Mo-2Sn-2Zr-2Nb-1.5Cr-0.1Si (TC21) alloy was studied. Additionally, an investigation of worn surface texture using the Abbott Firestone curve was conducted on MATLAB and Gwyddion software. For 20 min, the samples were heated to 925 °C. They were then cooled at different rates using water quenching (WQ), air cooling (AC), and furnace cooling (FC). Following that, the samples were aged for four hours at 600 °C. The microstructure consists of retained β-phase (βr), secondary α-phase (α_s_), and primary α-phase (α_p_). The α_s_-phase precipitated within the β_r_-phase in the case of aged samples and solutions treated with AC. The results show that the highest ultimate compressive strength of 1997 MPa was obtained for the FC + Aging and the sample owing to the existence of a high-volume fraction of α_p_-phase in the structure. On the other hand, the lowest ultimate compressive strength of 1473 MPa was achieved for the AC sample due to having a high amount of fine α_s_-phase. Comparing the WQ and WQ + Aging conditions, it is clear that the WQ + Aging condition shows 51% enhancement in wear property due to the presence of the α_s_-phase after applying the aging process. On the other hand, the wear properties of AC and FC after the aging process improve by only 18% and 4%, respectively. There is a slight variation in the friction coefficient values after the solution treatment process at different cooling rates (WQ, AC & FC). However, after applying the aging process, there is a clear change in WQ + Aging and AC + Aging conditions, where the value of the friction coefficient decreases by 19% and 12%, respectively. For the Abbott Firestone technique, the forged sample has a lower percentage of exploitation zone (77%) and a larger percentage of high peaks (22%). On the contrary, FC and FC + Aging samples have a higher percentage of exploitation zone (81%) and a lower percentage of high peaks (17%) compared to other samples.

## Introduction

Because of their low density, superior room and raised temperature strength, and exceptional resistance to fatigue, titanium-based alloys are desirable metallic materials for aircraft and have been widely used in the aerospace, automotive, and biomedical industries. However, these alloys were said to have famously poor wear characteristics because of their low working hardening, resistance to plastic shearing, and protection from surface oxide generated as a result of high temperatures (caused by frictional heating) during dry sliding. The low thermal conductivity of titanium alloys is one of the key causes of their poor tribological characteristics. Titanium alloys exhibit poor tribological properties, including a high friction coefficient, limited abrasion resistance, and severe adhesive wear^1–4^. Numerous heat treatment procedures can give titanium alloys the common microstructure of equiaxed, lamellar, and duplex structures. ɑ-phase is known to exhibit equiaxed, lamellar, and acicular geometric morphologies. Equiaxed microstructures serve better high-strength and ductility applications. Both fatigue and creep properties appear to be resistant to lamellar microstructures^5–7^.

TC21 (also known as Ti-6Al-2Sn-2Zr-1Cr-3Mo-2Nb-0.1Si) titanium alloy is a new type of ɑ+β category that has exceptional mechanical strength and fracture toughness. This alloy has undergone extensive research on several topics, including microstructure changes, heat treatments, and mechanical properties, since it is a potential material for structural components in aircraft and vehicle applications. Additionally, TC21 alloy is taken into consideration for other technical applications, including engine valves, gears, shaft components, and piston pins, where friction and wear performance must also be taken into consideration. The disadvantages of TC21 alloy, which are shared by other titanium alloys, include a large friction coefficient, a low strain-hardening coefficient, and poor shear strength, among others, which severely restrict its use in tribological disciplines. Only a few studies on fretting wear have been done on the TC21 alloy in the tribological areas, compared to the most commonly used Ti-6Al-4 V alloy. Many elements of wear characteristics, such as dry sliding wear at ambient temperature and above, mild-to-severe wear transition, the evolution of near-surface microstructure, and surface damage mechanisms, are yet unknown^8–12^.

Particularly for titanium-based alloys, the severe wear transition is crucial because, while mild wear behavior is frequently regarded as safe, severe wear behavior is undesirable from the perspective of technical applications. The most often discussed topic up to this point has been the production of tribo-oxide layers and their impact on the wear transition of titanium alloys. The formation of tribo-oxide layers on the worn surfaces of the Ti6Al4V and Ti6.5Al3.5Mo1.5Zr0.3Si alloys has frequently been observed to greatly slow the transition to severe wear at ambient temperature^13–15^. However, the underlying mechanism driving the transition from mild to severe wear without producing tribo-oxide layers on the surfaces of titanium-based alloys has not yet been identified. Therefore, a thorough examination of these factors is required. It also helps to assess the tribological application and adopt directional steps to enhance the wear characteristics of TC21 alloy^16,17^.

Ibrahim et al.^18^, the influences of applying various heat treatment processes on microstructure, compression, and wear properties of Ti6.55Al3.41Mo1.77Zr alloy. They found that the optimal combination of compression and wear resistance was achieved by heat treating at 1050 °C to obtain a fine lamellar structure. Fathy et al.^19^ investigated the effects of thermal oxidation on the wear behavior of TC21 alloy. They found that the best wear resistance had been achieved for specimens oxidized at 800 °C. Elshaer and Ibrahim^20^ investigated the effect of cold deformation with 15% reduction in height and heat treatments on the microstructure and wear properties of TC21 alloy. They discovered that the fine structure of β-phase that was obtained immediately after 15% deformation had a significant effect in raising the hardness of the deformed specimens and subsequently improving the wear behavior. Therefore, the goal of this work was to investigate how hot forging and heat treatments affected the TC21 alloy’s microstructure, cold compression, and tribological properties in detail. Additionally, worn surface textures were studied using MATLAB and Gwyddion software based on Abbott Firestone techniques.

## Material and experimental procedures

### Material

The current TC21 alloy was supplied by Baoji Hanz Material Technology Co., Ltd., Shanxi, China, as cast material in a cylindrical shape of 120 mm in diameter and 190 mm in length. For the hot forging process, a wire cutting machine was used to cut the TC21 sample, which had dimensions of 30 mm in diameter and 110 mm in length. The hot forging process was applied at 700 °C to decrease the diameter of the cast bar from 30 mm to 10 mm. Then, the sample was machined to reduce the diameter to 8 mm. Stress relief annealing was used for two hours at 550 °C, followed by furnace cooling, to lessen the remaining stress during hot-forging. The chemical composition of the analyzed as-cast TC21 alloy, as determined using a Foundry Pro-master spectrometer (OXFORD device, Germany), is presented in Table 1. To measure the transformation temperatures during continuous heating, a horizontal pushrod dilatometer with computer control (LINSEIS DIL L76 instrument, Germany) was employed. For a dilatation test, a sample with dimensions of 5 mm in diameter and 20 mm in length was machined using a CNC wire cutting machine. The sample was heated to 1100 °C in static air at a rate of 10 °C/min while being held in contact with the pushrod. It was then allowed to cool in the air to room temperature. The change in specimen length as a function of temperature was recorded using the WIN-DIL software. Based on experiments, the transformation temperature (also known as the β transus temperature) was found to be around 960˚C.

**Table 1:** Chemical composition of analyzed TC21 alloy.

Nominal composition	Average chemical composition, Wt.%												
	Al	Mo	Zr	Sn	Nb	Cr	Si	Fe	C	*N*	H	O	Ti
TC21 alloy	6.51	2.94	2.23	2.09	1.97	1.57	0.09	0.20	0.01	0.01	0.001	0.07	Bal.

### Experimental procedures

**Fig. 1:**
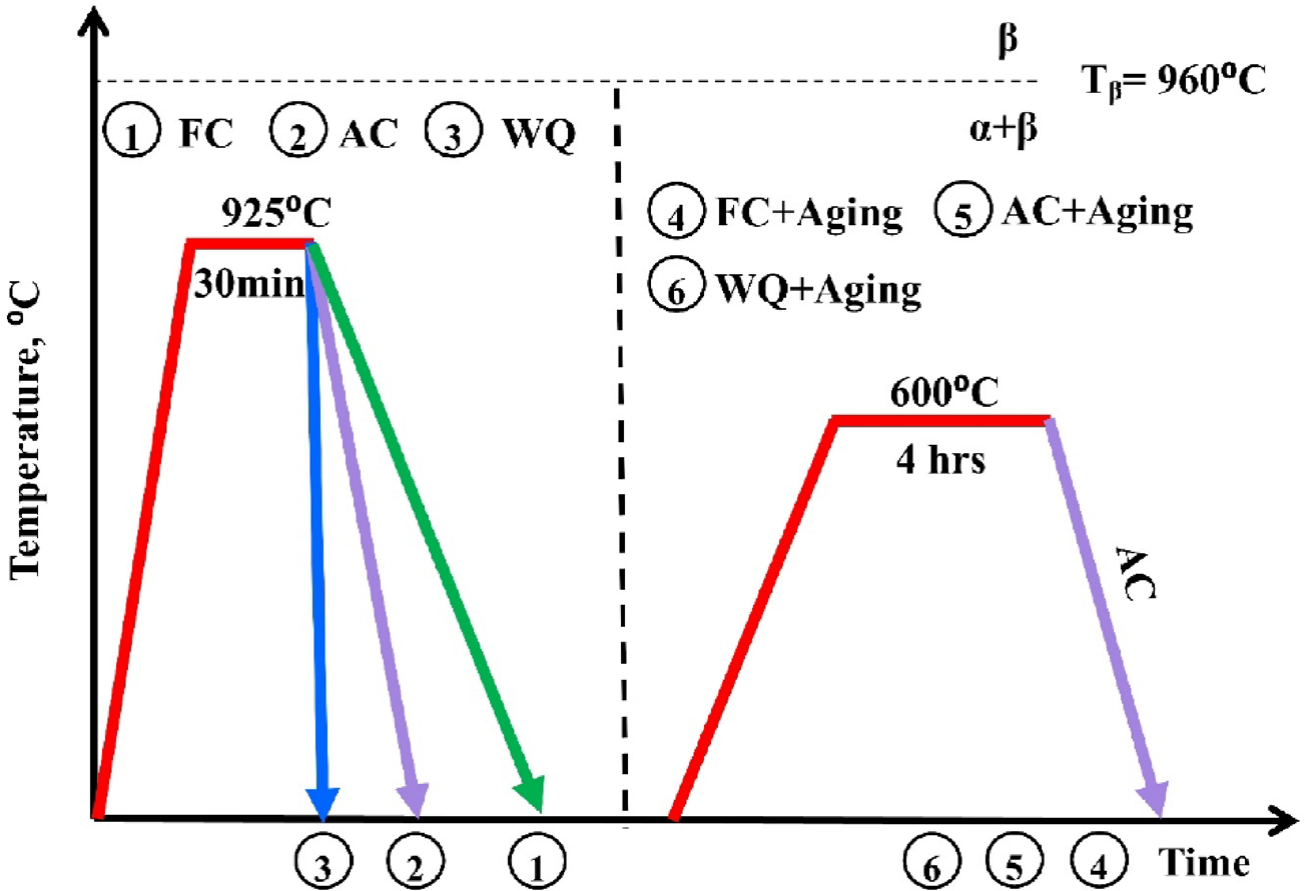
Schematic of the heat treatment cycles for TC21 alloy.

The furnace utilized for all heat treatment cycles was programmable and had a regulated environment with a maximum temperature of 3000 °C. To reduce the titanium-oxygen interaction, all heat treatment cycles were conducted in an argon atmosphere. The sample was also placed in the center of the furnace under all heating conditions, and a calibrated K-type thermocouple was installed there to ensure that the sample reached the proper temperatures. Figure 1 shows the schematic of the heat treatment cycles for TC21 alloy. The β transformation temperature, also known as the β transus (T_β_) temperature, was determined before the heat treatment cycles used in this work were chosen. This alloy had a measured β transus temperature of about 960 °C. It was designed to apply the solution treatment at the α + β phase region (below T_β_). Thus, for 20 min, the samples were exposed to a solution treatment at 925 °C. They were subsequently cooled to room temperature at a variety of rates, including water quenching (WQ), air cooling (AC), and furnace cooling (FC). The samples were then aged for 4 h at 600 °C, followed by AC. By conventional techniques, samples were ground, polished, and etched (3 ml of HF, 30 ml of HNO_3_, and 67 ml of H_2_O).

Vickers hardness tester apparatus (model 5030 SKV, England) was used to evaluate the hardness using a load of 10 kg for 15 s in compliance with the ASTM E92-23 Standard. Five readings for each sample were averaged and noted. A universal testing machine (model WDW-300, China) was used to perform the compression test at room temperature with a crosshead speed of 0.5. Compression testing was performed on samples with dimensions of 8 mm in diameter and 12 mm in length according to ASTM E9-12. The average of the results from three different samples was used to acquire data on all compression properties. The resistance to wear was measured at room temperature using a pin-on-disk wear tribometer testing instrument (T-01 M). A cylindrical pin sample with dimensions of 8 diameter and 12 mm in length was placed against a revolving disc of tool steel (65 HRC). The wear test was conducted with a continuous sliding distance of 120 m, a sliding speed of 0.2 m/s, a normal load of 80 N, and a time of 10 min. The samples were cleaned both before and after the test, and their weights were measured using an electronic scale with 0.1 mg accuracy. For each condition, the average wear rate was calculated across three tests. The friction force was measured during the test using a data acquisition system saved in a PC, and the value of the friction force was then divided by the applied force (80 N) to calculate the friction coefficient. A field emission scanning electron microscope (FESEM) was used to examine the microstructure and worn surface. Photographs of microstructures and worn surfaces have been graphically processed using MATLAB and Gwyddion software.

## Results and discussion

### Dilatometer behavior

**Fig. 2:**
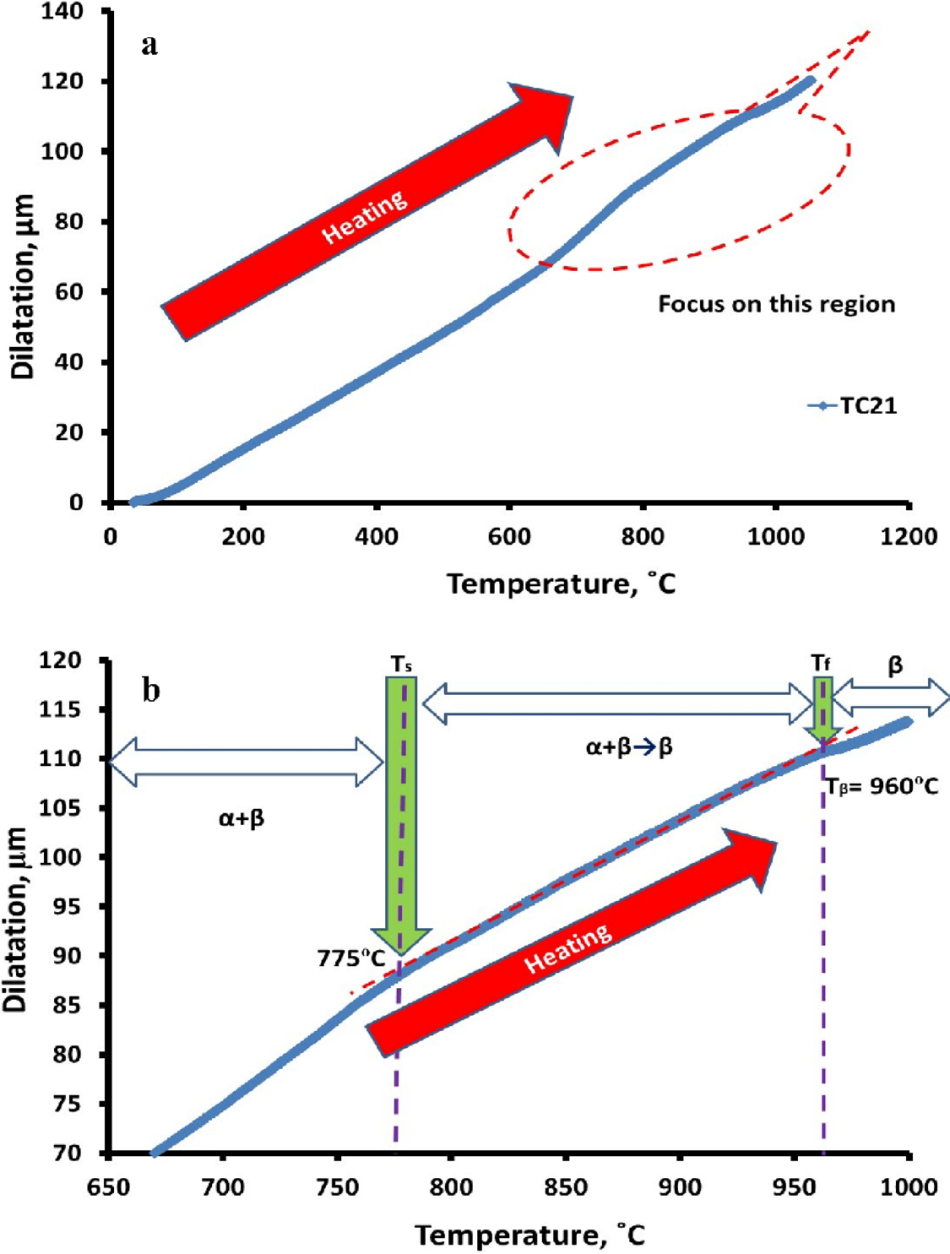
Dilatometric heating curve of TC21 alloy (**a**) overall view and (**b**) start and final temperatures of α + β→β phase change.

The general dilatometric curve was produced by continually heating the TC21 sample; see Fig. 2a. Transformation temperatures are computed using the slope variations in the curve that correlate to changes in sample length.

The sample continues to shrink as the α + β→β phase change proceeds, reaching 960 °C (Fig. 2b). The sample length starts to grow as the temperature rises. As seen in Fig. 2b, the sample shrinks further throughout the α + β→β phase transformation before coming to a stop at 960 °C. On the dilatometric curve, the sample length will increase relative to T_β_ (960 °C). If the temperature is increased above the transus temperature, T_β_ (960 °C), a complete β-phase occurs. The dilatometric curve mentioned above shows two different reflection locations as the heating temperature rises. The beginning transformation temperature of α + β→β and the final transformation temperature are represented by the letters (T_s_) and (T_f_), respectively. For the TC21 alloy under investigation, T_s_ was recorded at 775 °C and T_f_ at 960 °C.

### Microstructure

**Fig. 3:**
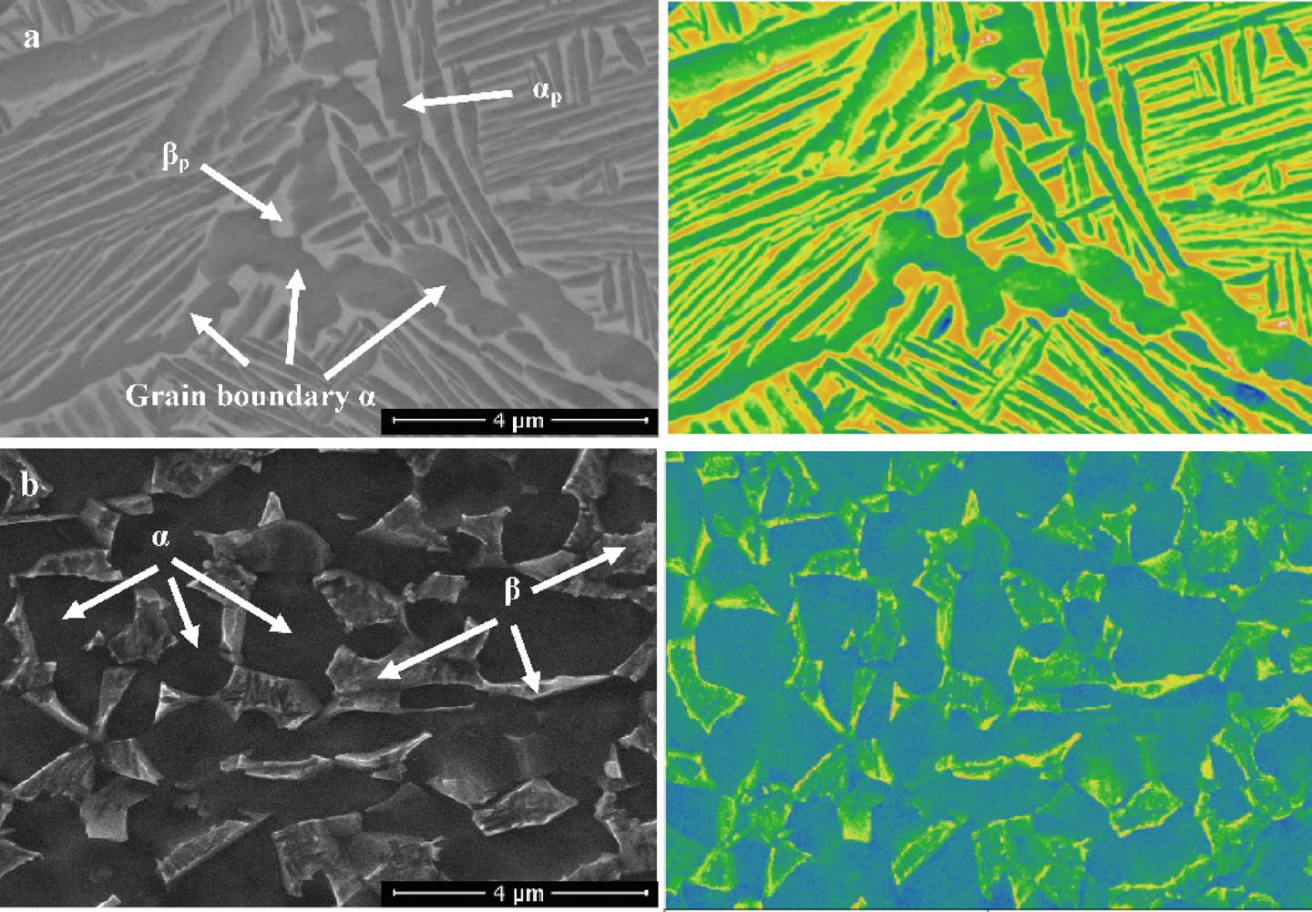
Micrographs of **(a)** as-cast, and **(b)** forged samples.

Figure 3a shows the as-cast microstructure of the TC21 titanium alloy, which had a coarse equiaxed β-phase and different α-phase morphologies. The α-phase was found along the grain boundaries and in the matrix between the β-phase. However, Fig. 3b displays the forged microstructure of the TC21 alloy, which had a fine equiaxed microstructure with primary equiaxed α-phase (α_p_), a dark area, and transformed β-phase (β_trans_), a bright area. The distribution of α_p_-phase in β_trans_-phase was homogeneous.

**Fig. 4:**
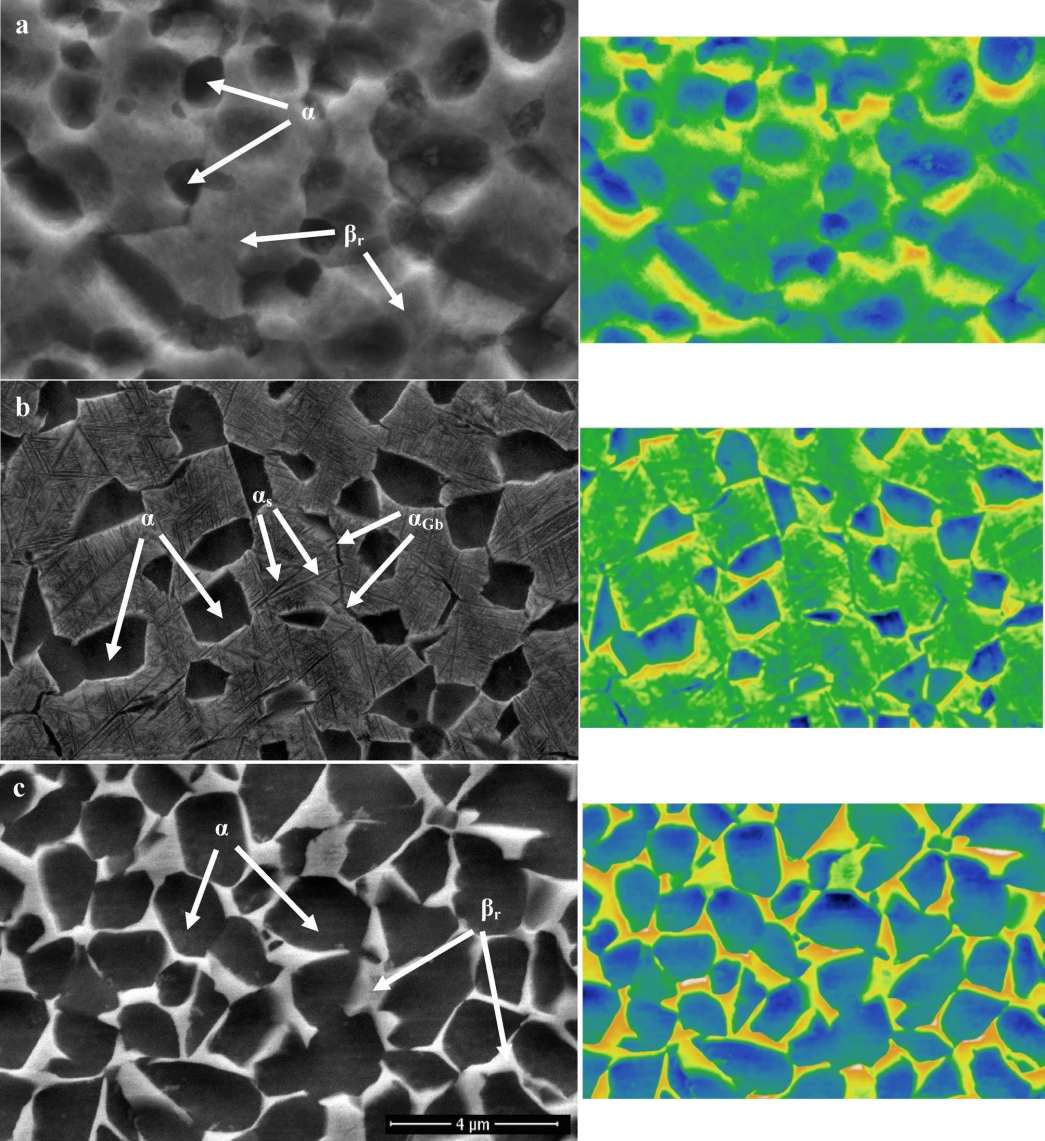
Micrographs of (**a**) WQ, (**b**) AC, and (**c**) FC samples.

Figure 4 depicts the microstructure of TC21 samples that were heated to 925 °C and then cooled using three distinct cooling rates: WQ, AC, and FC. All samples have primary α-phase (α_p_) and retained β-phase (β_r_) in their microstructure, with secondary α-phase (α_s_) in the case of the AC sample (Fig. 4b). α_s_-phase did not precipitate in the WQ sample, leading to substantial supersaturation due to stabilizing element diffusion inside the β_r_-phase. Thus, these components will stop α_s_-phase precipitation from occurring during β_r_-phase. Because of this, there is no chance for α_s_-phase precipitation within β_r_-phase (Fig. 4a). Additionally, as seen in Fig. 4c, the very slow cooling rate of the FC sample prevented the α_s_-phase from precipitating because there was insufficient driving force for α_s_-phase nucleation. Naturally, the FC sample didn’t produce adequate super-saturation; therefore, there was no α_s_-phase precipitation within β_r_-phase^21^.

**Fig. 5:**
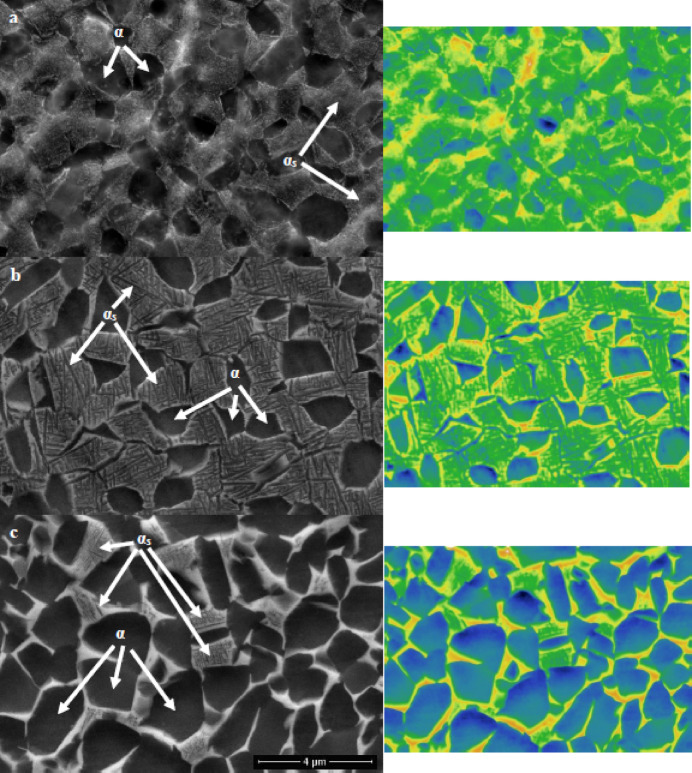
Micrographs of (**a**) WQ + Aging, (**b**) AC + Aging, and (**c**) FC + Aging samples.

Figure 5 illustrates how solution treatment factors (temperature & cooling rate) significantly impact the microstructure of aged samples. The morphology of α_p_ and β_r_-phases remained the same throughout the aging process, in contrast to the solution-treated one. Nevertheless, under all conditions of cooling rates, this process results in changes in volume fractions and particle size of the α_p_ and β_r_-phases, as well as precipitation in the α_s_-phase. Therefore, α_s_-phase precipitated in all conditions of aged samples (Fig. 5). The primary reason for the strengthening of TC21 alloy is due to these precipitations of α_s_-phase within β_r_-phase^22^. In conclusion, solution-treated samples of TC21 alloy that were heated to 925 °C, followed by AC, as well as aged samples, caused the α_s_-phase to precipitate within the β_r_-phase. Although there were variations in the heat treatment cycles, these results partially concurred with those of Elshaer and Ibrahim^20^.

### Hardness

**Fig. 6:**
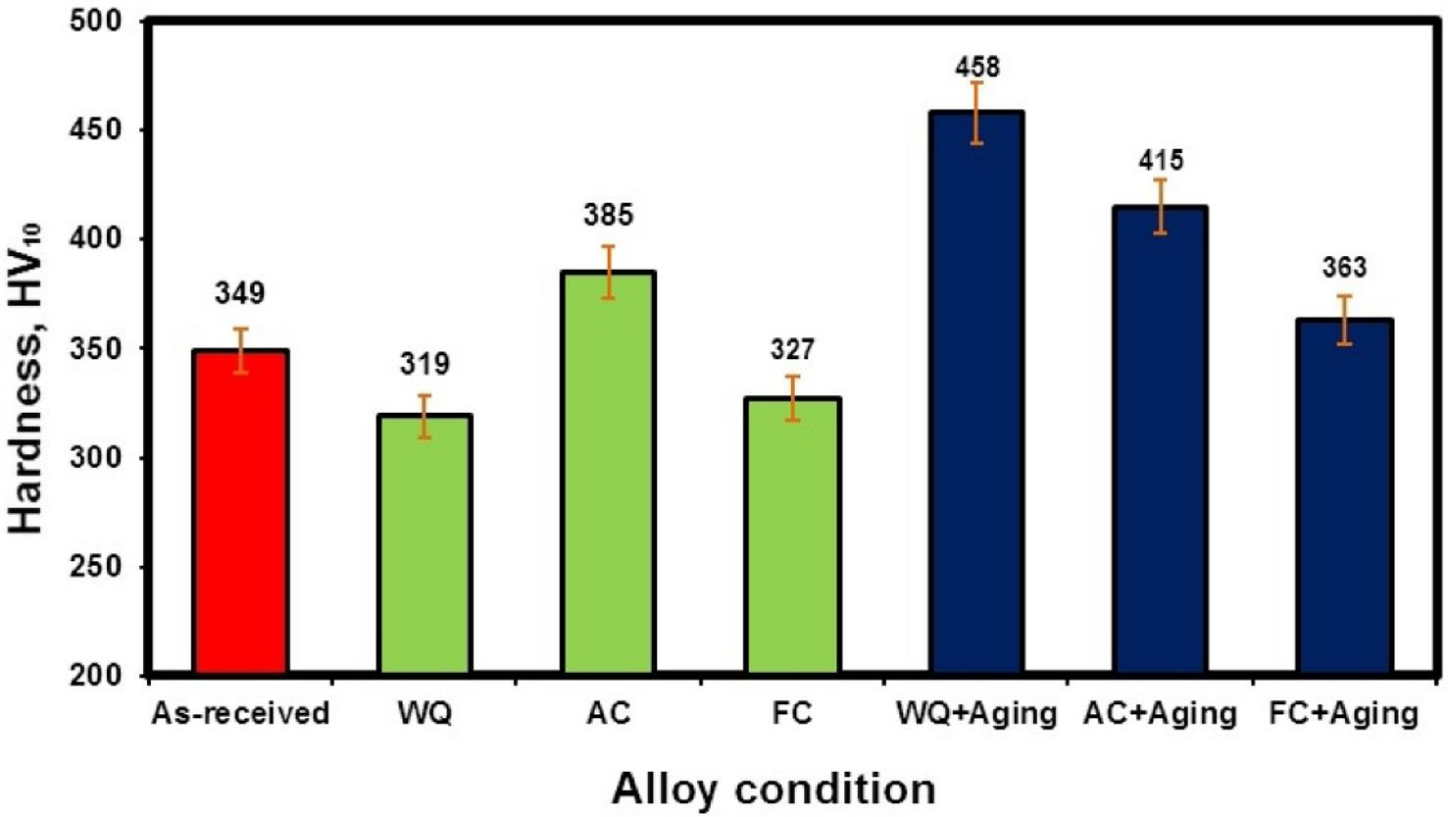
Hardness of the TC21 alloy for forged and heat-treated conditions.

Figure 6 illustrates the difference in hardness of forged, solution-treated samples at different cooling rates and after aging. The hardness of the forged sample is 350 HV_10_. The FC and WQ samples’ hardness values dropped during solution treatment when compared to the forged sample. The lowest hardness value was 319 HV_10_, which was found in the WQ sample. In contrast to the WQ sample, the FC sample had a higher hardness of 327 HV_10_. Furthermore, because of the precipitate of α_s_-phase within β_r_-phase, the AC sample showed the maximum hardness of 385 HV_10_ when compared to the other cooling rates. Both the FC + Aging and AC + Aging samples had hardness values of 363 and 415 HV_10_, respectively. The WQ + Aging sample had the highest hardness value, 458 HV_10_, because of the high amount of β_r_ and αs-phase precipitation, as well as the low amount of α_p_-phase, in comparison to the others.

### Cold compressive strength

**Fig. 7:**
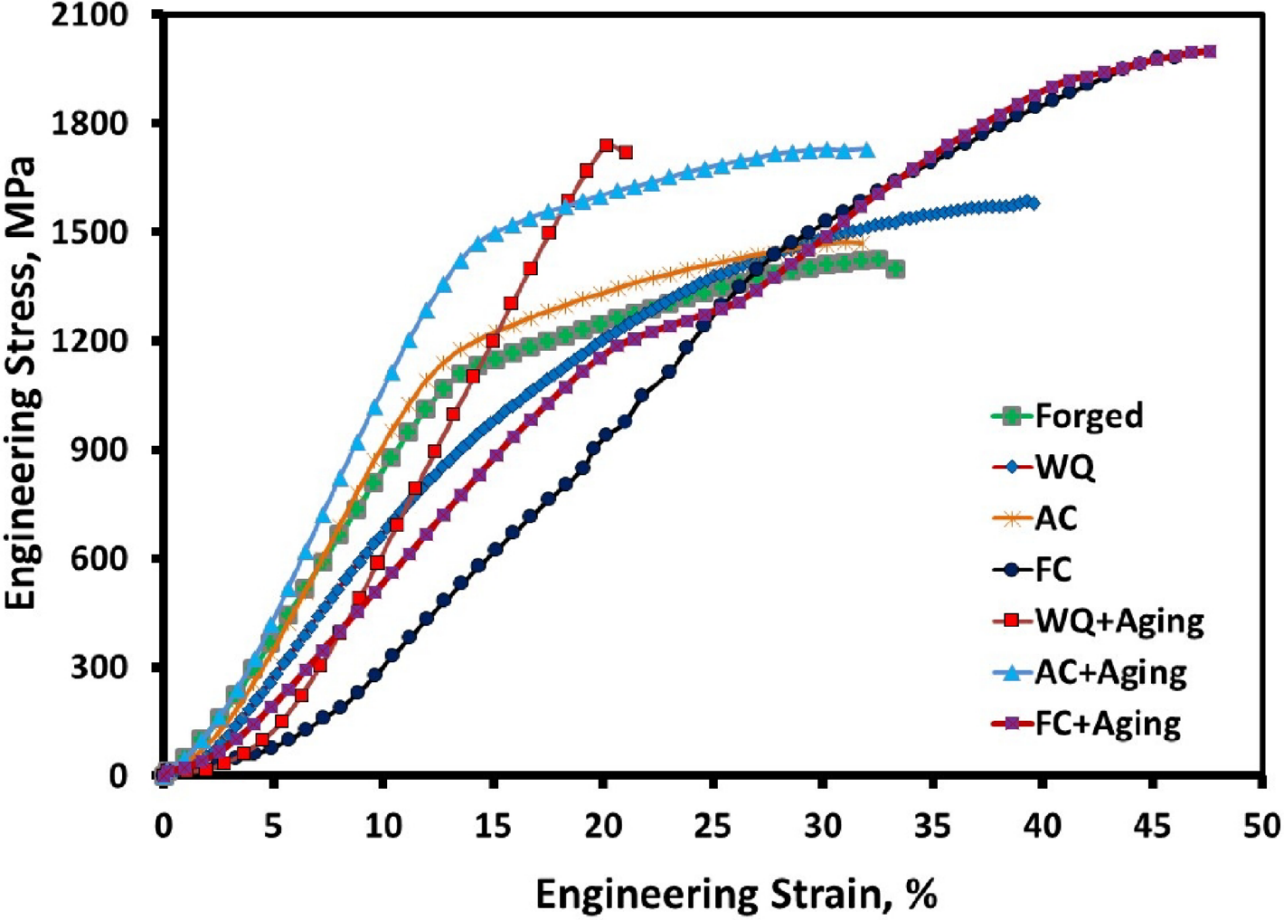
Engineering stress-strain curves of compression test for different conditions.

The engineering stress-strain curves for forged, solution-treated with various cooling rates, and aged samples are shown in Fig. 7. The ultimate Compressive strength is affected by solution treatment with different cooling rates. The results revealed that the highest ultimate compressive strengths of 1979 and 1997 MPa were obtained for the FC and FC + Aging samples owing to the existence of a high-volume fraction of α_p_-phase in the structure, and they also have the largest reduction in height, which was 33 and 32%, respectively. On the other hand, the lowest ultimate compressive strength of 1473 MPa was achieved for the AC sample, and the WQ + Aging sample has the lowest reduction in height of 12% due to having a high amount of fine α_s_-phase. Table 2 lists the ultimate compressive strength of the analyzed TC21 alloy.

**Table 2:** Compression properties of the analyzed TC21 alloy.

Conditions	Ultimate compressive strength, MPa					Yield compressive strength, MPa					Reduction in height, %				
	Reading			Average	Standard deviation	Reading			Average	Standard deviation	Reading			Average	Standard deviation
Forged	1672	1668	1659	1666	6.66	1062	1075	1070	1069	6.56	26	28	27	27	1
WQ	1590	1587	1579	1585	5.69	888	894	885	889	4.58	31	28	32	30	2.08
AC	1469	1470	1480	1473	6.08	1090	1088	1097	1092	4.73	26	27	25	26	0.82
FC	1977	1975	1985	1979	5.29	1285	1295	1287	1289	5.29	35	33	31	33	2
WQ + Aging	1732	1741	1742	1738	5.51	1669	1675	1663	1669	6	12	11	14	12	1.53
AC + Aging	1726	1725	1736	1729	6.08	1416	1420	1429	1422	6.66	22	21	20	21	0.82
FC + Aging	1995	1992	2003	1997	5.69	1192	1182	1181	1185	6.08	33	31	31	32	1.16

### Tribological properties

#### Wear rate behavior

**Fig. 8:**
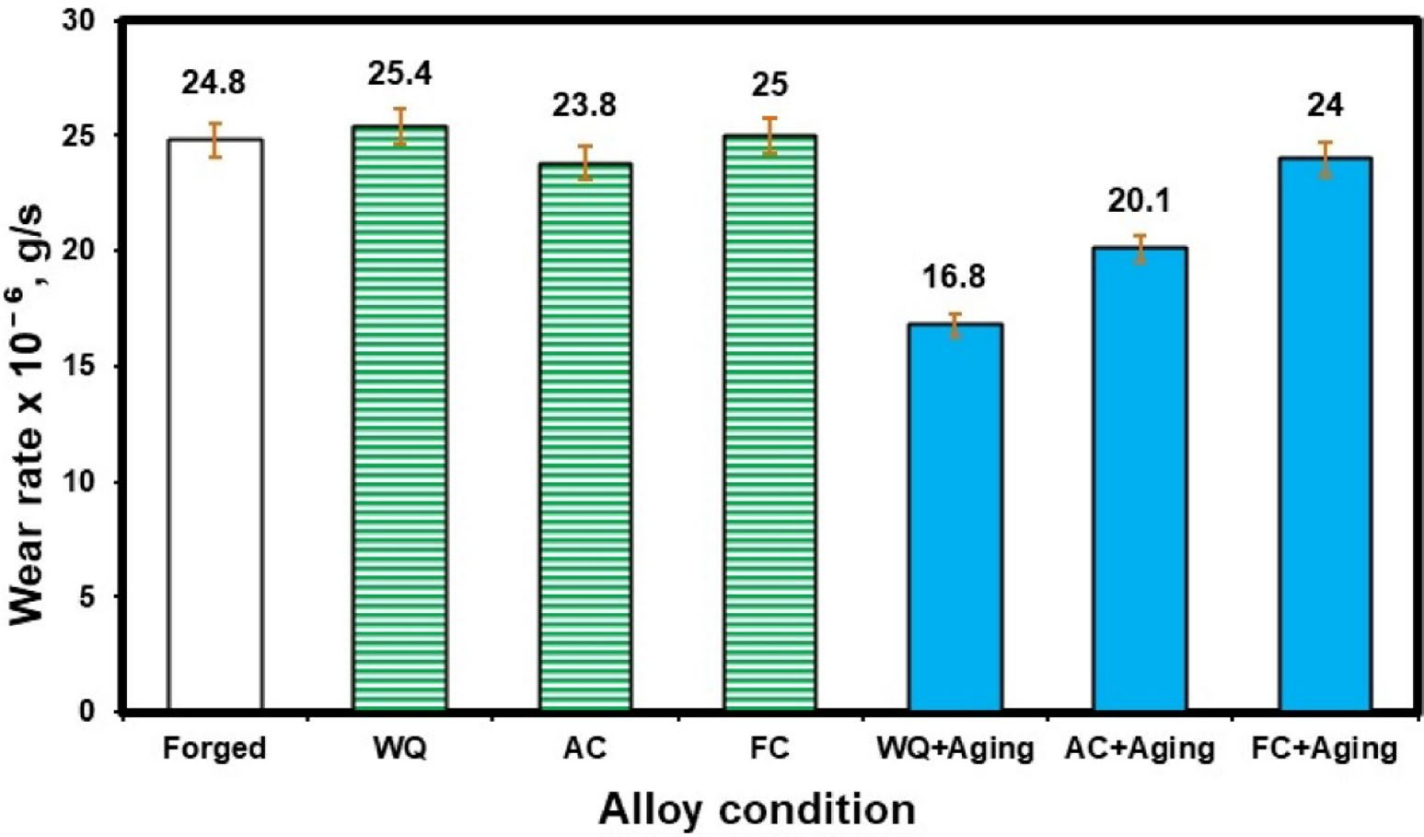
Wear rate of the TC21 alloy for different conditions.

Figure 8 displays the wear rates for forged, WQ, AC, FC, WQ + Aging, AC + Aging, and FC + Aging conditions. Each value represents the average of three specimens. The three main factors influencing wear performance are temperature, microstructure, and hardness^23–25^. There are two main reasons why titanium alloys have subpar tribological properties, identified by Molinari et al.^26^: (1) poor plastic shearing resistance and work-hardening threshold, (2) very little protection from surface oxide because of the high flash temperature caused by friction during wear. The wear rate for the forged samples was 24.8 × 10^− 6^ g/s. After solution treatment, AC samples recorded the lowest wear rate of 23.8 × 10^− 6^ g/s. This could be because of the existence of the α_s_-phase (secondary α-phase). However, because of the significant amount of the α_p_-phase, the WQ samples showed the highest wear rate of 25.4 × 10^− 6^ g/s when compared to forged, AC, and WQ samples.

According to Archard’s law, the hardness and wear rate of a material are inversely related^27^. This suggests that as hardness increases, a material’s wear rate reduces. Given that the hardness values varied significantly under the current conditions, the experimental sliding wear data demonstrated a strong association with Archard’s law. The wear rate of WQ + Aging condition was found to rise as a result of their higher hardness (458 HV_10_) in comparison to the other conditions. Due to their great hardness, the WQ + Aging condition exhibited the lowest recorded wear rate of 16.8 × 10^− 6^ g/s when compared to all other conditions. Thus, the microstructural elements or hardness of the samples under investigation may be able to control the rate of wear.

In general, a harder material may hold a thicker oxide coating more securely than a softer one^28^. This suggests that the more resilient the WQ + Aging condition, the more it may be able to sustain an oxide layer with a higher critical thickness before flaking off. The removal, reformation, and additional thickening of the oxide layer will alter the rate of wear. The AC + Aging condition observed a wear rate of 20.1 × 10^− 6^ g/s. However, the AC condition had a higher wear rate of 23.8 × 10^− 6^ g/s because it had a low percentage of precipitated α_s_ phase in comparison to the AC + Aging condition. Comparing the WQ and WQ + Aging conditions, it is clear that the WQ + Aging condition revealed 51% enhancement in wear property due to the presence of the α_s_-phase after applying the aging process. On the other hand, the wear properties of AC and FC after the aging process improve by only 18% and 4%, respectively. Therefore, the aging process after the FC has little effect on reducing the wear rate. A reduced wear rate was observed in the aged samples compared to the solution-treated ones, as the actual area of contact directly affects the wear rate. In summary, applying the aging process after rapid cooling by water (WQ) has a much greater impact on reducing the wear rate for TC21 alloy than intermediate cooling by air (AC) and slow cooling by furnace (FC). This is explained by the secondary α-platelets that precipitated inside the β-rubs. This finding agrees with the study by Elshaer and Ibrahim^20^; nevertheless, there were some differences in the temperatures of the aging and solution treatment processes.

#### Friction coefficient

**Fig. 9:**
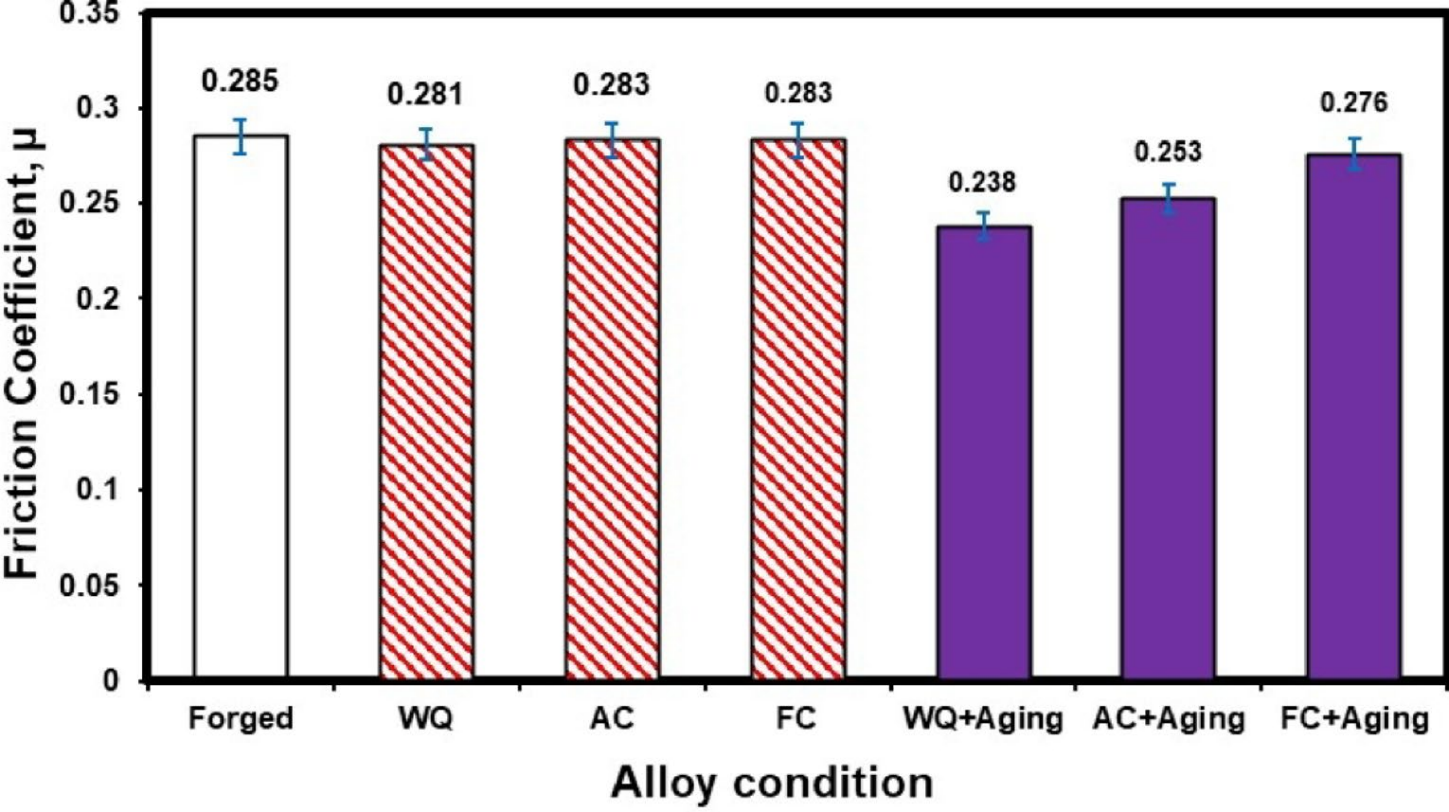
Friction coefficient of the TC21 alloy at different conditions.

Figure 9 displays the friction coefficient of the TC21 titanium alloy under various conditions. There is a slight variation in the friction coefficient values after the solution treatment process at different cooling rates (WQ, AC & FC). However, after applying the aging process, there is a clear change in the WQ + Aging and AC + Aging conditions, where the value of the friction coefficient decreases by 19% and 12%, respectively. Therefore, applying the aging process after the solution treatment at different cooling rates is very important for the forged TC21 samples to decrease the friction coefficient values.

#### Worn surfaces and Abbott Firestone curves

**Fig. 10:**
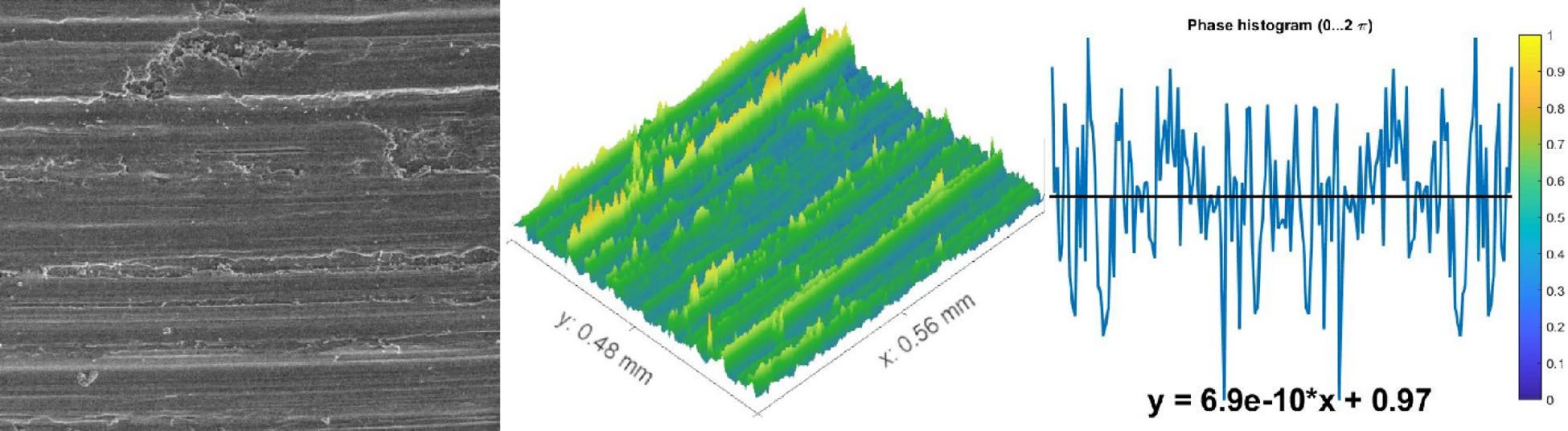
Worn surface of the forged sample.

**Fig. 11:**
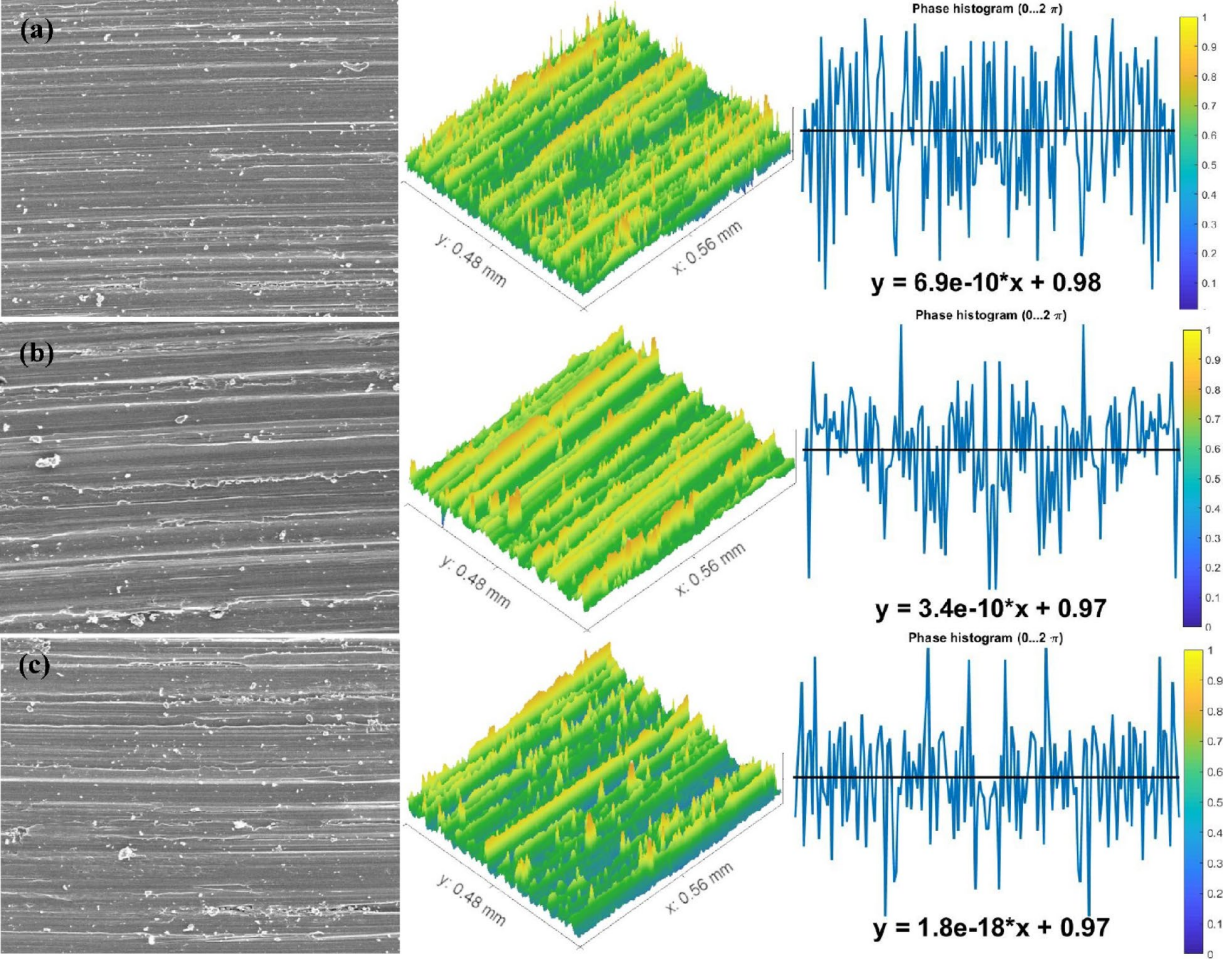
Worn surface of the (**a**) WQ, (**b**) AC, and (**c**) FC samples.

**Fig. 12:**
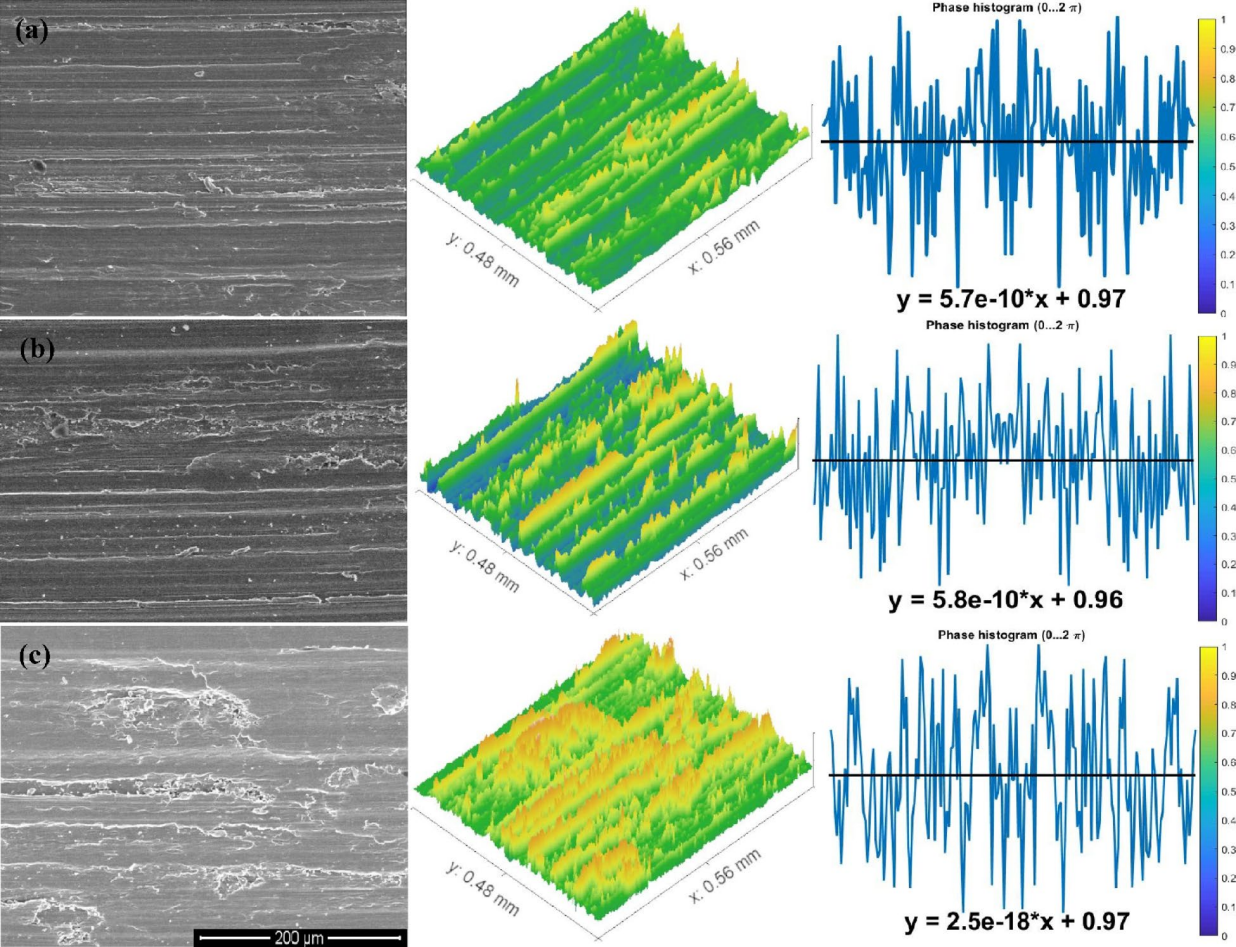
Worn surface of the (**a**) WQ + Aging, (**b**) AC + Aging, and (**c**) FC + Aging samples.

The above results were in good agreement with the morphological study of wear fragments. Some chosen samples of TC21 alloy under various conditions displayed characteristically worn surface morphologies in the FESEM micrographs in Figs. 10, 11 and 12. In every studied sample, some abrasion wear was found. The majority of evaluated samples show some indications of plastic deformation over the worn surfaces. Over the worn tracks, there are constant sliding markings with scratches or grooves that are plastically distorted. The wear track demonstrated a micro-fragmentation process for different situations. Additionally, TC21 alloy displayed typical adhesive traces and abrasive furrows under various conditions. Except for WQ + Aging, which has higher hardness, all conditions contain delamination and grooves. Additionally, some oxide particles and debris were seen at WQ + Aging and FC + Aging conditions in Fig. 12a, c. Nevertheless, as seen in Fig. 10, the forged condition displayed extensive scratches over the worn surface. The wear findings shown in Fig. 8 validated these worn surface features. The worn surface of FC samples for the solution-treated sample group had many wear mechanisms, as indicated in Fig. 11c. These mechanisms include micro-cutting, delamination, and deep scratches caused by plastic deformation. This is because the worn surface has gotten softer.

### Determination of surface roughness by Abbott Firestone curves

**Fig. 13:**
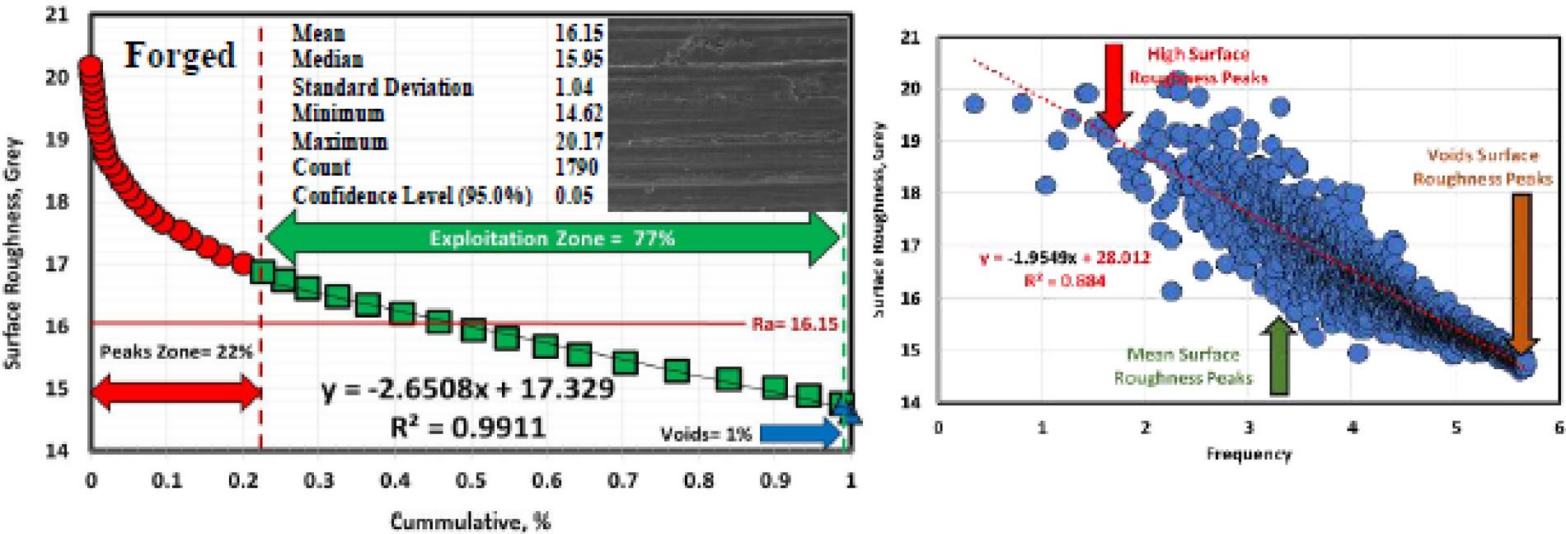
Abbott Firestone curve of the worn surface texture for the forged sample.

**Fig. 14:**
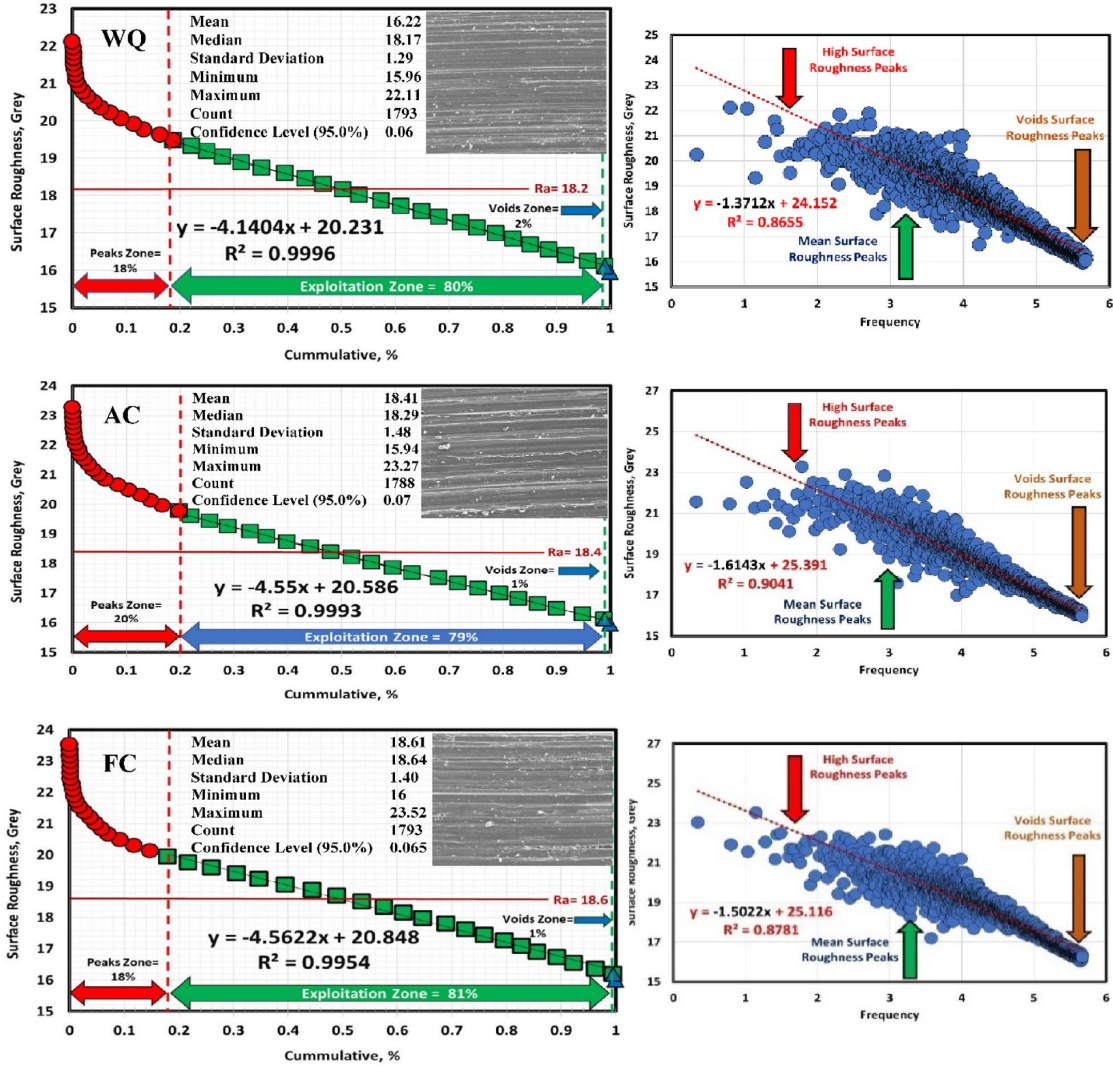
Abbott Firestone curve of the worn surface textures for WQ, AC, and FC samples.

**Fig. 15:**
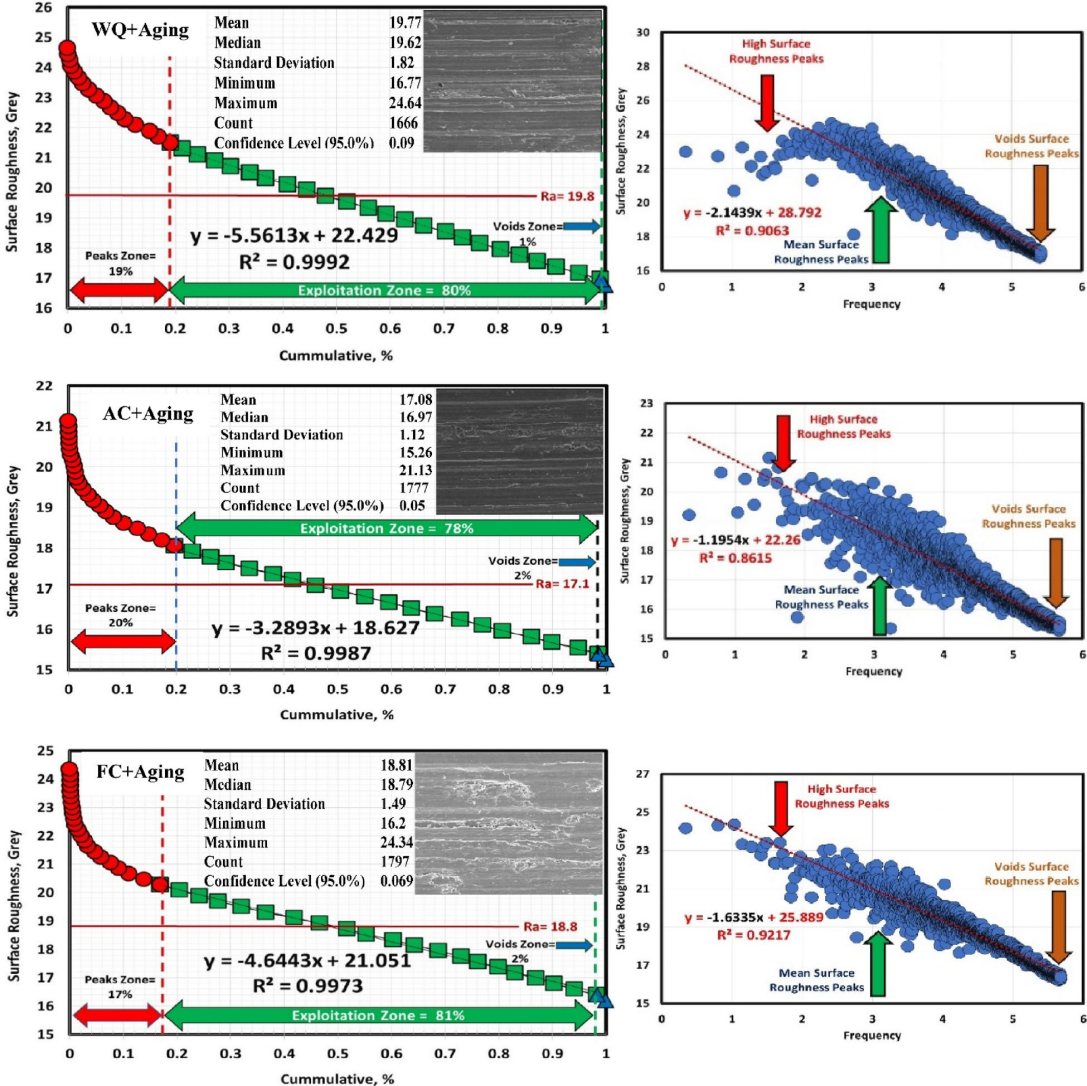
Abbott Firestone curve of worn surface texture for WQ + Aging, AC + Aging, & FC + Aging samples.

An effective technique for assessing surface texture characterizations is the Abbott Firestone curve, which can be used to identify the high peaks, exploitation, and void zones of aviation parts such as engines, landing gear, flap tracks, and fuselage sections. The safety of aircraft components is known to be significantly increased by expanding the exploitation zone, and the lubrication system is said to be somewhat enhanced by the existence of voids. Measurement and assessment of surface roughness have advanced dramatically in recent years, both quantitatively and qualitatively. Thankfully, quantitative findings may be obtained by computing surface texture from FESEM micrographs of the worn surface using the MATLAB software. A statistical analysis of the MATLAB software’s computation results was conducted. Figures 13, 14 and 15 demonstrate Abbott Firestone curves and surface textures of worn surfaces under various conditions. The forged sample has a lower percentage of the exploitation zone (77%) and a larger percentage of high peaks (22%). On the contrary, FC and FC+Aging samples have a higher percentage of exploitation zone (81%) and a lower percentage of high peaks (17%) compared to other samples. The exploitation zone yields a high percentage for every sample, whereas the void zone yields a comparatively low percentage. The void zone in all samples is within the range of 1 to 2%. Wemay therefore conclude that low cooling rate by furnace cooling (FC) and aging process after furnace cooling (FC+Aging) can increase the exploitation zone and lower the high peaks zone.

## Conclusions

The impact of forging and heat treatment processes on cold compressive and tribological properties (wear rate & friction coefficient) of Ti-6Al-3Mo-2Sn-2Zr-2Nb-1.5Cr-0.1Si (TC21) alloy was studied. In addition, an investigation of worn surface texture by the Abbott Firestone curve was conducted on MATLAB and Gwyddion software. Several conclusions can be drawn from this analysis, including the following:

1. Solution-treated samples of TC21 alloy that were heated to 925 °C, followed by AC, as well as aged samples, caused the α_s_-phase to precipitate within the β_r_-phase.
2. The highest ultimate compressive strength of 1997 MPa was obtained for FC + Aging and the sample owing to the existence of a high-volume fraction of α_p_-phase in the structure. On the other hand, the lowest ultimate compressive strength of 1473 MPa was achieved for the AC sample due to having a high amount of fine α_s_-phase.
3. Applying the aging process after rapid cooling by water (WQ) has a much greater impact on reducing the wear rate for TC21 alloy than intermediate cooling by air (AC) and slow cooling by furnace (FC).
4. The friction coefficient values after the solution treatment process at different cooling rates (WQ, AC & FC) slightly vary. However, after applying the aging process, there is a clear change in the WQ + Aging and AC + Aging conditions, where the friction coefficient value decreases by 19% and 12%, respectively.
5. A higher percentage of high peaks (22%) and a smaller percentage of exploitation zone (77%) are present in the forged sample. Conversely, compared to other samples, the FC and FC + Aging samples show a lower percentage of high peaks (17%) and a higher percentage of exploitation zone (81%).
6. FC and FC + Aging samples have higher exploitation and lower high peak zones.

## Data Availability

All data generated or analyzed during this study are included in this published article.
